# Scottish Index of Multiple Deprivation (SIMD) indicators as predictors of mortality among patients hospitalised with COVID-19 disease in the Lothian Region, Scotland during the first wave: a cohort study

**DOI:** 10.1186/s12939-023-02017-y

**Published:** 2023-10-05

**Authors:** Marcello S. Scopazzini, Roo Nicola Rose Cave, Callum P. Mutch, Daniella A. Ross, Anda Bularga, Margo Chase-Topping, Mark Woolhouse, Oliver Koch, DataLoch Covid-19 Collaborative, Atul Anand, Kathy Harrison, Ally Hume, Catriona Waugh, Catherine Stables, Chloe Brook, Chris Duncan, David Homan, Erin Cadger, Ioanna Lampaki, Jennifer Daub, Jilly McKay, Neil Murray, Ronnie Harkess, Shedrack Ezu, Sophie McCall, Stela McLachlan, Alastair Thomson, Alistair Stewart, Daniella Ene, Hazel Neilson, Juergen Caris, Maria McMenemy, Nazir Lone, Nicola Rigglesford, Paul Schofield, Sophie David, Stephen Young, Tracey McKinley, Tracey Rapson, Anna K. Jamieson, Arjuna A. Sivakumaran, Arun Parajuli, Ed Whittaker, Emma K. Watson, Ha Bao Trung Le, Hannah M. M. Preston, Jason Yang, John P. Kelly, Jonathan Wubetu, Julia Guerrero Enriquez, Kathryn A. W. Knight, Louisa R. Cary, Oscar C. N. Maltby, Rosie Callender, Sarah H. Goodwin, Thomas H. Clouston, Thomas J. McCormick, XinYi Ng, Zaina Sharif, Anoop Shah, Colan Mehaffey, Ken Lee, Laura Woods-Dunlop, Michael Gray, Nicholas Mills, Pamela Linksted, Peter Cairns, Rob Baxter, Stephen Lavenberg, Susan Buckingham, Meghan R. Perry, Claire L. Mackintosh

**Affiliations:** 1 Clinical Infection Research Group, NHS Lothian Infection Service, Western General Hospital, Edinburgh, UK; 2 London School of Hygiene and Tropical Medicine, London, UK; 3 The School of Biological Sciences, University of Edinburgh, Edinburgh, UK; 4 British Heart Foundation Centre for Cardiovascular Science, University of Edinburgh, Edinburgh, UK; 5 The Roslin Institute and Royal (Dick) School of Veterinary Studies, University of Edinburgh, Edinburgh, UK; 6 Usher Institute, University of Edinburgh, Edinburgh, UK; 7 DataLoch COVID-19 Collaborative, DataLoch, University of Edinburgh, Edinburgh, UK

**Keywords:** Deprivation, Covid-19, SIMD indicators, Mortality

## Abstract

**Background:**

Sars-CoV-2, the causative agent of COVID-19, has led to more than 226,000 deaths in the UK and multiple risk factors for mortality including age, sex and deprivation have been identified. This study aimed to identify which individual indicators of the Scottish Index of Multiple Deprivation (SIMD), an area-based deprivation index, were predictive of mortality.

**Methods:**

This was a prospective cohort study of anonymised electronic health records of 710 consecutive patients hospitalised with Covid-19 disease between March and June 2020 in the Lothian Region of Southeast Scotland. Data sources included automatically extracted data from national electronic platforms and manually extracted data from individual admission records. Exposure variables of interest were SIMD quintiles and 12 indicators of deprivation deemed clinically relevant selected from the SIMD. Our primary outcome was mortality. Age and sex adjusted univariable and multivariable analyses were used to determine measures of association between exposures of interest and the primary outcome.

**Results:**

After adjusting for age and sex, we found an increased risk of mortality in the more deprived SIMD quintiles 1 and 3 (OR 1.75, CI 0.99–3.08, *p* = 0.053 and OR 2.17, CI 1.22–3.86, *p* = 0.009, respectively), but this association was not upheld in our multivariable model containing age, sex, Performance Status and clinical parameters of severity at admission. Of the 12 pre-selected indicators of deprivation, two were associated with greater mortality in our multivariable analysis: income deprivation rate categorised by quartile (Q4 (most deprived): 2.11 (1.20–3.77) *p* = 0.011)) and greater than expected hospitalisations due to alcohol per SIMD data zone (1.96 (1.28–3.00) *p* = 0.002)).

**Conclusions:**

SIMD as an aggregate measure of deprivation was not predictive of mortality in our cohort when other exposure measures were accounted for. However, we identified a two-fold increased risk of mortality in patients residing in areas with greater income-deprivation and/or number of hospitalisations due to alcohol. In areas where aggregate measures fail to capture pockets of deprivation, exploring the impact of specific SIMD indicators may be helpful in targeting resources to residents at risk of poorer outcomes from Covid-19.

**Supplementary Information:**

The online version contains supplementary material available at 10.1186/s12939-023-02017-y.

## Introduction

Coronavirus disease 2019 (Covid-19) first emerged in December 2020, in Wuhan, China, and has now contributed to more than 226,000 deaths in the UK [1, 2]. Previous studies during the first wave identified being male and older, presence of comorbidities, and greater socio-economic deprivation at diagnosis as major risk factors for death and intensive care unit (ICU) admission [3–6].

Two large population-based studies – the OpenSAFELY Collaborative in England (June 2020) and the REACT-SCOT study in Scotland (October 2020) – have produced authoritative evidence that lower socio-economic status was associated with severe disease and mortality from Covid-19 even when adjusted for age, sex, and number of co-morbidities at presentation [3, 6]. Scotland, with the unenviable sobriquet “the Sick Man of Europe”, consistently ranks among the least healthy countries in Europe. Multi-generational poverty and social exclusion, drug- and alcohol-dependence, and poor educational attainment have proved pervasively difficult to eradicate and continue to negatively impact health outcomes [7, 8].

The Scottish Index of Multiple Deprivation (SIMD) ranks geographical areas of similar population across seven standardised domains (Income, Employment, Education, Health, Access to Services, Crime and Housing) to target interventions aimed at alleviating social inequalities [9, 10]. The SIMD ranks 6,976 geographical areas, termed datazones, derived from postcodes; SIMD is therefore a relative rather than absolute indicator of deprivation reflecting in-country geographical variation [10]. Studies conducted in the first and subsequent waves of Covid-19 have focused on how aggregate SIMD quintile rankings influence outcomes: patients in the lowest SIMD quintile had a consistently greater risk of death and Covid-19 has exacerbated healthcare inequalities across Scotland [4, 11, 12]. Studies looking at separate indicators of deprivation have shown that area-specific measures of income deprivation and overcrowding were predictive of poorer outcomes among affected residents, but, to date, other potentially relevant indicators within the SIMD have not been evaluated [13].

In this study of 710 patients hospitalised with Covid-19 in the Lothian Region of South-East Scotland between March 1^st^ and June 30^th^, 2020, we investigated the impact of 12 clinically relevant individual SIMD indicators and constructed a model to determine their relationship with mortality in this cohort.

## Methods

### Study setting and databases

Data sources were linked using the Community Hospital Index (CHI), a unique identifier for patients residing in Scotland. Data were automatically extracted from the following platforms: laboratory information management systems, the Scottish Morbidity Record, the Scottish Drug Dispensing Database, and the Scottish Care Information Store.

Clinical and demographic data obtained from individual hospitalisation events were manually linked by a team of researchers at the Western General Hospital (Edinburgh, UK). Primary reasons for admission to ICU and mortality were adjudicated by the clinical research team to determine if Covid-19 was the principal contributor. Prior to analysis, all data were anonymised and stored in a data repository (DataLoch, Edinburgh, United Kingdom).

### Participants

This was a prospective cohort study which included any patients aged > 18 whose listed postcode was in one of East Lothian, City of Edinburgh, Midlothian, or West Lothian councils and who were admitted to hospital with a laboratory confirmed, positive polymerase chain reaction (PCR) test for SARS-CoV-2 between 01/03/20 and 30/06/2020.

### Variables

Our primary outcome was mortality, defined as all-cause mortality occurring among patients admitted to hospital with a positive PCR for SARS-CoV-2 during the study period, in line with definitions described in other UK-based cohort studies [3, 6].

The primary exposures of interest were Scottish Index of Multiple Deprivation (SIMD) indicators drawn from publicly available records. The SIMD is an aggregate measure of deprivation that comprises seven domains (Income, Employment, Education, Health, Access to Services, Crime and Housing) further subdivided into 37 component indicators. All indicators are interdependent with varying levels of between-indicator association. The SIMD ranks 6,976 datazones across Scotland into a relative ranking of area-based deprivation (Scottish Index of Multiple Deprivation (SIMD) 2020, version 2, Government of Scotland) [9, 10]. The study team selected 12 indicators considered clinically relevant for final analysis (see Table 1).

**Table 1:** Indicators of Scottish Index of Multiple Deprivation (2020 version 2) deemed clinically relevant to analysis

**SIMD Indicator**	**Description**
Income Deprivation Rate	Percentage of residents who are income deprived, per datazone
Employment Deprivation Rate	Percentage of residents who are employment deprived, per datazone
Comparative Illness Factor	Age and sex standardised ratio of observed and expected number of recipients of disability allowance, per datazone
Hospital stays related to alcohol use	Age and sex standardised ratio of observed and expected hospital admissions with alcohol-related conditions, per datazone
Hospital stays related to drug use	Age and sex standardised ratio of observed and expected hospital admissions with drug-related conditions, per datazone
Standardised Mortality Ratio	Age and sex standardised ratio of observed and expected all-cause death, per datazone
Proportion of population prescribed drugs for anxiety, depression, and/or psychosis	Estimated proportion of residents, per datazone
Emergency stays in hospital	Age and sex standardised ratio of observed and expected emergency room hospital visits, per datazone
Proportion of working age population with no higher qualifications	Proportion of residents, per datazone
Drive to GP	Average driving time to nearest GP surgery, in minutes
Public Transport to GP	Average travel time by public transport to nearest GP surgery, in minutes
Overcrowding rate	Percentage of households that are overcrowded, per datazone

Additional exposure variables were selected from the risk factors associated with mortality identified in a separate descriptive cohort study in the same group of patients [5]. These included: demographic variables of age, sex, and ethnicity; World Health Organization (WHO) Performance Status, which categorises the impact of chronic disease severity on patient activity levels (0 = able to carry out normal activity without restriction; 1 = restricted in strenuous activity but ambulatory; 2 = ambulatory for > 50% of waking hours; 3 = symptomatic in a chair or bedridden for > 50% of waking hours; and 4 = completely disabled); admission pulse, in beats per minute; admission haemoglobin, in grams per Litre; neutrophil and lymphocyte counts (cells × 10^5^); creatinine level, in milligrams/decilitre; and SIMD quintile.

### Statistical analysis

Continuous outcomes were categorised into standardised brackets with the normal reference range used as the reference variable (admission pulse, admission haemoglobin, admission neutrophil and lymphocyte counts) whilst age was categorised into age groups with age group 50–59 used as the reference variable, in line with the ISARIC4C and OpenSAFELY cohort studies [3, 6]. Continuous SIMD indicator variables were categorised into quartiles, with the lowest quartile (least deprived) used as the reference variable. For SIMD standardised ratios, the variable was categorised as greater than or less than expected occurrence (standardised ratio) in each datazone.

**Fig. 1:**
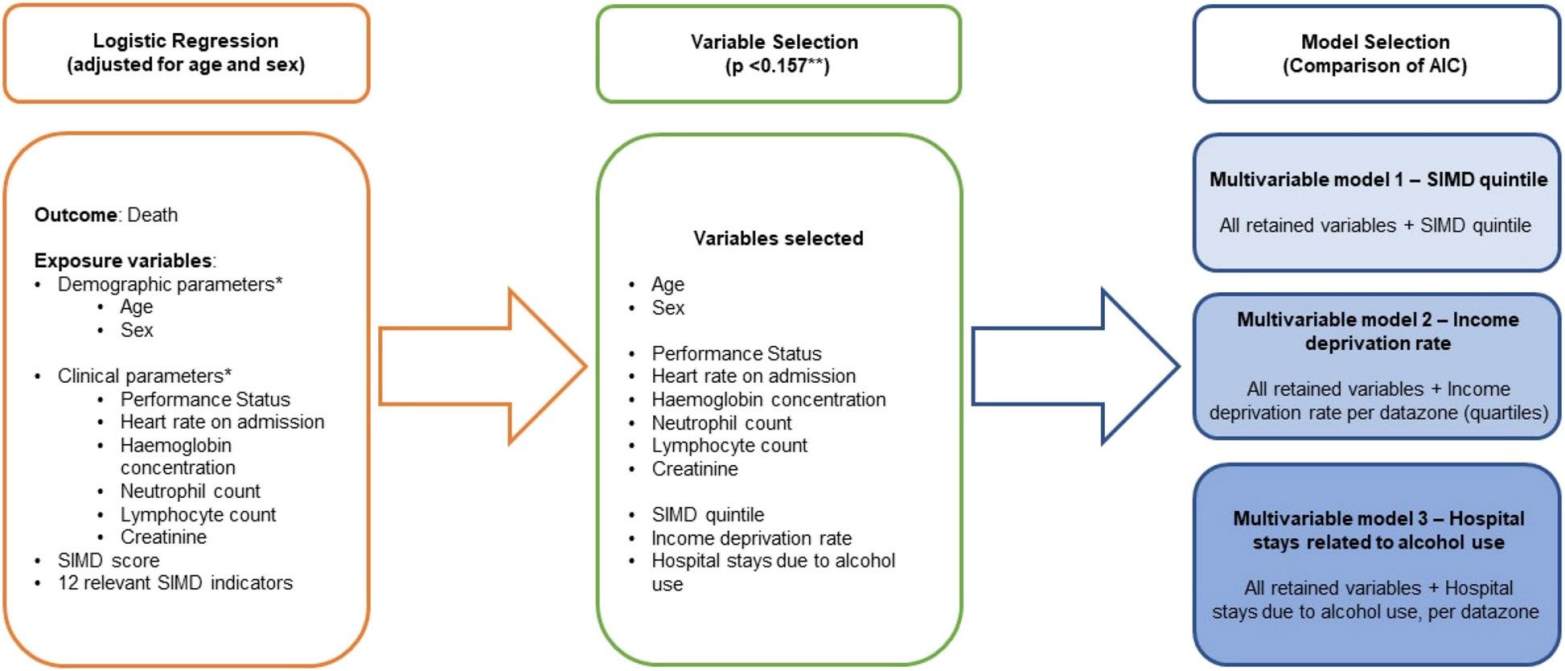
Step-by-step representation of statistical analyses employed in the study. Legend: *Demographic and Clinical Parameters determined from Mutch et al. 2022 [5] “Performance status: A key factor in predicting mortality in the first wave of COVID-19 in South-East Scotland”. Demographic parameters = Age (in years), sex; Clinical parameters = Performance Status (WHO Standardized Categories); Admission pulse rate (in beats/minute); Haemoglobin concentration (in grams/Litre); Neutrophil count (cells × 10^5^); Lymphocyte count (cells × 10^5^); Creatinine (in milligrams/decilitre). SIMD score = quintile distribution; 12 relevant SIMD indicators = Income Deprivation Rate; Employment Deprivation Rate; Comparative Illness Factor; Hospital stays related to alcohol use; Hospital stays related to drug use; Standardised Mortality Ratio; Proportion of population prescribed drugs for anxiety, depression, and/or psychosis; Emergency stays in hospital; Proportion of working age population with no higher qualifications; Drive to GP (in minutes); Public Transport to GP (in minutes); Overcrowding rate (*Scottish Index of Multiple Deprivation (2020, version 2)*). ** Variables had *P* < 0.157 in the univariable analysis. *P* < 0.157 selected as a screening value appropriate for subsequent multivariable model selection by Akaike Information Criterion (AIC) with a study population of 710. (*Timo and Ilkka 1986; Perez-Guzman et al. 2021*)

Detailed description of analyses and modelling are further described in Fig. 1.

All 12 SIMD indicators selected by the study team were then assessed for the strength of between-indicator association, and association with age and sex using Cramer’s V [14].

Univariable analysis using logistic regression was carried out on age and sex to confirm the previously observed association with mortality (see Supplementary data). As a result, the remaining univariable models, including the 12 selected indicators of deprivation (Table 1), Performance Status and clinical parameters at admission, were run with age and sex included. Variables from these adjusted models with a *P*-value of < 0.157 were carried forward into a multivariable model. A screening *P*-value of < 0.157 is recommended for a sample size of 710 [15–17]. Because of the expected strong association between income deprivation rate by quartile and number of admissions due to alcohol use per datazone (Cramer V: 0.54), they could not be included in the same model. We therefore developed three nested models: SIMD quintile (Model 1), income deprivation rate by quartile (Model 2) and number of admissions due to excess alcohol per datazone (Model 3) and the model fit was assessed using Akaike information criterion (AIC). The model with the lowest AIC was considered the best.

We carried out a post hoc sensitivity analysis rerunning Model 1, using Lothian-specific SIMD quintile distributions. Results from both Lothian-specific and nationally-derived SIMD quintile distributions were compared.

### Approvals

Ethical approval was granted by the Lothian NHS Board (reference number CG/DF/2087). Linkage of anonymous datasets was performed by DataLoch, a data driven initiative designed to provide a secure repository of health and social care data in Southeast Scotland (Usher Institute, University of Edinburgh). Access to the final database was restricted to the core team of researchers with specific approvals and only accessible via a secure NHS network.

## Results

### Baseline characteristics, symptoms, and clinical parameters at presentation

Between March 1^st^ and June 30^th^, 2020, 726 patients were admitted to one of three hospitals in the Lothian Region (East Lothian, Midlothian, City of Edinburgh, and West Lothian councils) with a positive PCR for SARS-CoV-2. We excluded 13 patients from our analysis whose residential postcode was not within the Lothian Region, and three patients who did not have a registered postcode.

Case distribution by SIMD quintile was bimodal, with peaks in the second most deprived quintile (SIMD 2: *n* = 183, 25.8%) and least deprived quintile (SIMD 5: *n* = 190, 26.8%) but differences in case numbers between quintiles was negligible (see Table 2). Median age was 73 (IQR 58–83) and men accounted for 54.4% of patients. Age, sex, and ethnicity were similarly distributed across quintiles. Performance Status recorded at admission was graded at WHO stage 3 or more in just over a third of patients (34.4%, *n* = 245) and was evenly distributed across SIMD quintiles. Hypertension (*n* = 292, 41%), Diabetes (*n* = 166, 23%) and unspecified cancers (*n* = 144, 20%) were the most commonly reported co-morbidities, and most patients had two or more co-morbidities (*n* = 407, 57.3%) with minimal variation across SIMD quintiles. Symptoms and clinical parameters of severity at presentation were similar in patients with and without co-morbidities.

**Table 2:** Summary of anthropometric and clinical characteristics of patients included in study stratified by SIMD quintile

	**All Patients**	**1 = Most Deprived**	**2**	**3**	**4**	**5 = Least Deprived**
N, %	710	103 (14.5%)	183 (25.8%)	102 (14.4%)	132 (18.6%)	190 (26.8%)
Age on admission^a^ (years)	73 (58–83)	73 (60–82)	72 (57–81)	68 (53–81)	70.5 (54–82)	78 (64–86)
Sex						
Male	386 (54.4%)	50 (48.5%)	96 (52.5%)	55 (53.9%)	83 (62.9%)	102 (53.7%)
Female	324 (45.6%)	53 (51.5%)	87 (47.5%)	47 (46.1%)	49 (37.1%)	88 (46.3%)
Ethnicity						
White	551 (77.6%)	77 (74.7%)	140 (76.5%)	77 (75.5%)	107 (81%)	150 (78.9%)
Black, Asian, Minority Ethnic	28 (4%)	7 (6.8%)	10 (5.5%)	5 (4.9%)	< 5	< 5
Ethnicity not recorded	131 (18.4%)	19 (18.4%)	33 (18%)	20 (19.6%)	22 (16.7%)	37 (19.5%)
**Previous Health status**						
Performance status						
0	201 (28.3%)	24(23.3%)	42 (22.9%)	34 (33.3%)	44 (33.3%)	57 (30%)
1	131 (18.4%)	16 (15.5%)	46(25.1%)	18 (17.6%)	23 (17.4%)	28(14.7%)
2	130 (18.3%)	23 (22.3%)	38 (20.7%)	23 (22.5%)	22 (16.7%)	24 (12.6%)
3	200(28.1%)	34(33%)	47 (25.7%)	21(20.6%)	32 (24.2%)	66 (34.7%)
4	45 (6.3%)	6 (5.8%)	9 (4.9%)	6 (5.9%)	10 (7.6%)	14 (7.4%)
Missing	< 5	-	< 5	-	< 5	< 5
Co-morbidity count						
0	111 (15.6%)	15 (14.6%)	23 (12.6%)	22 (21.6%)	22 (16.7%)	29 (15.3%)
1	192 (27%)	24 (23.3%)	48 (26.2%)	25 (24.5%)	35 (26.5%)	60 (31.6%)
2 plus	407 (57.3%)	64 (62.1%)	112 (61.2%)	55 (53.9%)	75 (56.8%)	101 (53.1%)
Comorbidities						
Chronic Obstructive Pulmonary Disease	104 (15%)	21 (20%)	28 (27%)	11 (11%)	21 (20%)	23 (22%)
Diabetes Mellitus	166 (23%)	27 (16%)	57 (34%)	22 (13%)	31 (19%)	29 (18%)
Hypertension	292 (41%)	41 (14%)	78 (27%)	44 (15%)	55 (19%)	74 (25%)
Cancer	144 (20%)	19 (13%)	31 (22%)	15 (10%)	32 (22%)	47 (33%)
Other						
**Symptoms on presentation**						
Fever	428 (60%)	60 (58%)	110 (60%)	62 (60%)	85 (64%)	111 (58%)
Cough	478 (67%)	67 (65%)	119 (65%)	75 (73%)	89 (67%)	128 (67%)
Breathlessness	419 (59%)	59 (57%)	116 (63%)	55 (54%)	87 (66%)	102 (54%)
**Clinical parameters**^**a**^						
PaO2 (kilopascals)	10.5 (9.2, 12.8)	11.5 (9.2, 14.3)	10.9 (8.7, 12.8)	10.7 (9.2, 12.8)	10.5 (9.1, 12.8)	10.9 (9.2, 12.8)
Pulse (beats per minute)	91 (80, 105)	90 (80, 104)	90 (80, 103)	92 (82, 105)	94 (85, 109)	90 (76, 103)
Respiratory Rate (breaths/minute)	21 (18, 26)	20 (18, 24)	20 (18, 24)	22 (18, 28)	22 (20, 26)	20 (18, 24)

^a^Continuous variables are presented as median (Interquartile range). Categorical variables are presented as number (%)

### Outcomes

Outcomes were available for all 710 patients included in the study (see Table 3). All-cause mortality was recorded in 28% (*n* = 197) of patients; deaths were proportionately higher in patients in the most deprived quintile compared to the least deprived (SIMD 1–31% vs SIMD 5–26.8%) and fewer patients received mechanical ventilator support in the most deprived quintile compared to the least deprived (SIMD 1–41.6% v SIMD 5–65.2%). Data pertaining to suitability for intensive care and/or mechanical ventilation were not available for this study. Mean length of hospital stay was similar across SIMD quintiles.

**Table 3:** Outcomes for patients included in analysis stratified by SIMD quintile

		**All patients**	**1 = Most deprived**	**2**	**3**	**4**	**5 = least deprived**
	N	710	103	183	102	132	190
**Outcome**							
Dead	N (%)	197 (28%)	32 (31%)	47 (25.7%)	31 (30.4%)	36 (27.2%)	51 (26.8%)
Admission to ICU	N (%)	103 (15%)	12 (11.6%)	29 (15.8%)	19 (17.6%)	20 (15.1%)	23 (12%)
Required mechanical ventilation	N (%)	68 (66%)	5 (41.6%)	19 (65.5%)	13 (68.4%)	16 (80%)	15 (65.2%)
Length of hospital stay (in days)	Median (IQR)	8 (3, 19)	7 (3, 16)	9 (3, 22)	9 (4, 19)	8 (3, 19)	9 (3, 19)

### Correlation between clinically relevant indicators of deprivation and mortality

**Fig. 2:**
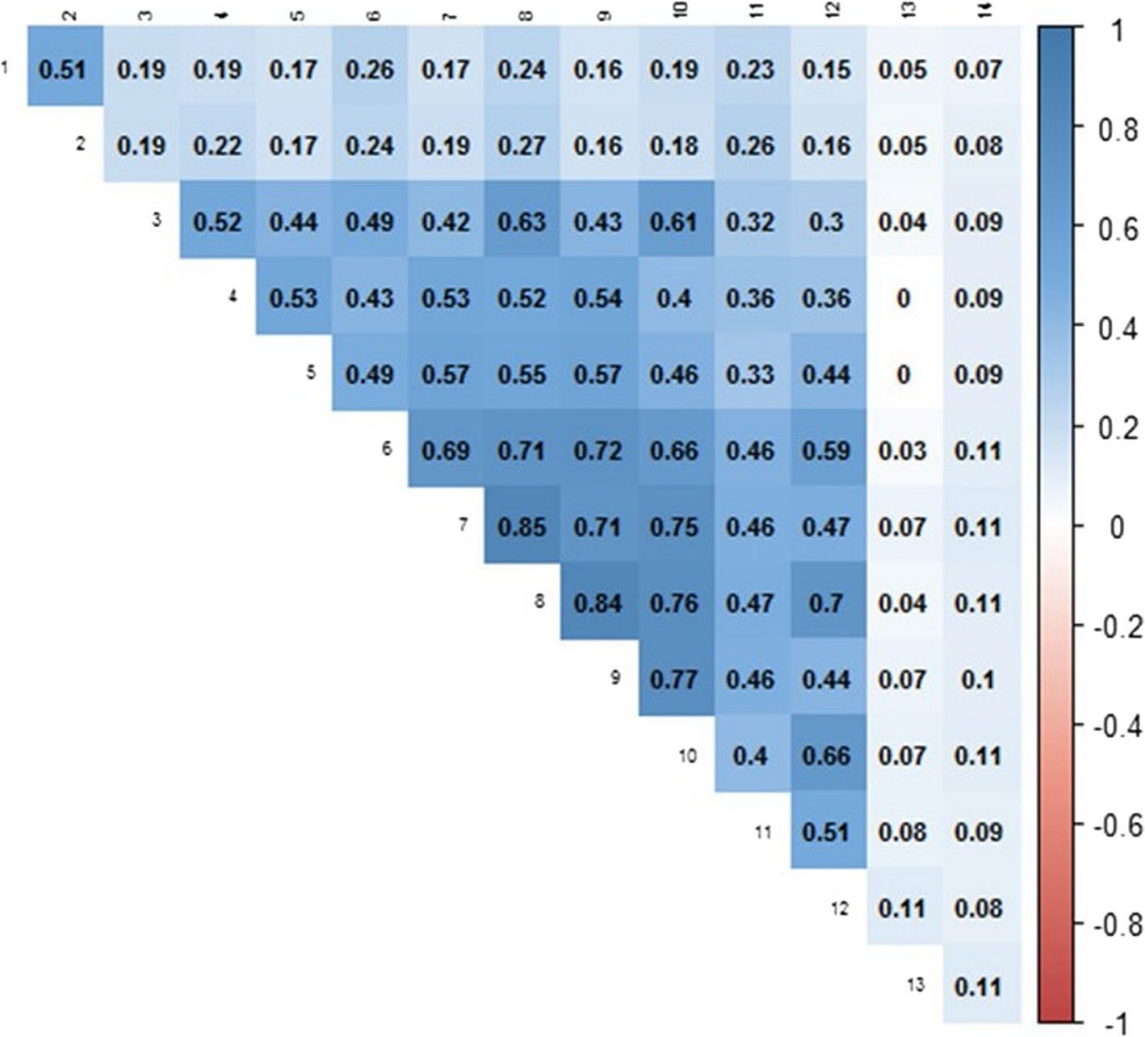
Cramer’s V Correlation Matrix of 12 selected indicators of deprivation plus age and sex. Deeper shading indicates higher degree of association. Legend: 1. Distance to nearest GP surgery per datazone, in minutes; 2. Distance to nearest GP surgery by public transport per datazone, in minutes; 3. Overcrowding rate; 4. Hospitalisations due to alcohol per datazone; 5. Hospitalisations due to drug use per datazone; 6. Emergency hospitalisations per datazone; 7. Employment rate; 8. Comparative Illness factor; 9. Income rate; 10. 16–19 year-olds without qualifications; 11. Standardised Mortality Ratio; 12. Prescriptions for anxiety, depression or psychosis per datazone; 13. Sex; 14. Age

To account for expected association between the 12 selected SIMD indicators selected for outcome analysis, a Cramer’s V correlation matrix was created which demonstrated a high degree of association between individual SIMD indicators (see Fig. 2). Relevant to our models, no SIMD indicators had medium or high association to age or sex.

### Associations between clinically relevant indicators of deprivation and mortality

Increasing age, male sex, and poorer Performance Status at diagnosis were all associated with mortality in our unadjusted and adjusted univariable logistic regression model.

Two SIMD quintiles were associated with higher mortality in the age- and sex-adjusted, univariable model (Quintile 1 (most deprived) *P* = 0.102, Quintile 3 *P* = 0.032)).

In our univariable analysis of the 12 clinically relevant area-based indicators of deprivation, two were significantly associated with mortality: income deprivation rate per datazone, categorised into quartiles (Quartile 4 (most deprived) *P* = 0.007) and greater than expected hospital stays due to alcohol use per datazone (*P* = 0.009) (see Table 4).

**Table 4:** Univariable age- and sex-adjusted logistic regression analysis for the association between mortality and exposure variables

**Exposure Variable *(SI units)***	**Range**	**Univariable Age and Sex-adjusted** **Odds Ratio (95% CI) and *P* value**
Performance Status, *(WHO standard categories)*	0	-
	1	2.24 (1.08–4.78) *P* = 0.033
	2	3.75 (1.78–8.24) *P* = < 0.001
	3	3.74 (1.81–8.09) *P* = 0.001
	4	7.11 (2.85–18.30) *P* < 0.001
Heart rate on admission *(beats per minute)*	60–99	-
	< 60	1.29 (0.44–3.67) *P* = 0.628
	> 99	1.92 (1.28–2.88) *P* = 0.002
Haemoglobin concentration *(grams/Litre)*	> 129	-
	< 100	2.92 (1.50–5.80) *P* = 0.002
	100–129	0.82 (0.54–1.27) *P* = 0.331
Neutrophil count *(cells* × *10*^*5*^*)*	2–7.5	-
	< 2	2.26 (1.10–4.59) *P* = 0.025
	> 7.5	1.61 (1.07–2.43) *P* = 0.022
Lymphocyte count *(cells* × *10*^*5*^*)*	> 1.4	-
	< 0.5	1.77 (0.96–3.30) *P* = 0.071
	0.5–1.4	0.83 (0.50–1.39) *P* = 0.476
Creatinine *(milligrams/decilitre)*	< 125	-
	> 125	2.46 (1.63–3.70) *P* < 0.001
SIMD quintile	5 (least deprived)	
	4	1.35 (0.78–2.33) *P* = 0.284
	3	1.91 (1.06–3.45) *P* = 0.032
	2	1.24 (0.75–2.05) *P* = 0.393
	1 (most deprived)	1.61 (0.91–2.85) *P* = 0.102
Income deprivation rate per datazone *(categorised by quartile)*	Q1 (least deprived)	-
	Q2	2.00 (1.17–3.46) *P* = 0.018
	Q3	1.70 (0.99–2.96) *P* = 0.056
	Q4 (most deprived)	2.05 (1.22–3.51) *P* = 0.007
Employment rate *(categorised by quartile)*	Q1 (least deprived)	-
	Q2	0.91 (0.51–1.61) *P* = 0.756
	Q3	1.41 (0.87–2.29) *P* = 0.168
	Q4 (most deprived)	1.26 (0.80–2.00) *P* = 0.315
Comparative Illness Factor (*Standardised Ratio*)	< expected	-
	> expected	1.24 (0.86–1.78) *P* = 0.250
Hospital admissions per datazone related to alcohol use (*Standardised Ratio*)	< expected	-
	> expected	1.68 (1.12–2.48) *P* = 0.009
Hospital admissions per datazone related to drug use (*Standardised Ratio*)	< expected	-
	> expected	1.33 (0.92–1.93) *P* = 0.126
Standardised mortality ratio (*Standardised Ratio*)	< expected	-
	> expected	1.09 (0.77–1.57) *P* = 0.616
Proportion of population being prescribed drugs for anxiety, depression or psychosis per datazone *(categorised into quartiles)*	Q1 (least deprived)	-
	Q2	0.97 (0.57–1.67) *P* = 0.908
	Q3	1.47 (0.86–2.55) *P* = 0.162
	Q4 (most deprived)	0.93 (0.54–1.61) *P* = 0.793
Standardised ratio of emergency stays in hospital (*Standardised Ratio*)	< expected	-
	> expected	1.29 (0.89–1.87) *P* = 0.182
Working age people with no qualifications (*Standardised Ratio*)	< expected	-
	> expected	0.97 (0.67–1.40) *P* = 0.886
Average drive time to a General Practitioner (GP) surgery in minutes *(categorised into quartiles)*	Q1 (least deprived)	-
	Q2	0.87 (0.53–1.41) *P* = 0.567
	Q3	1.10 (0.68–1.80) *P* = 0.692
	Q4 (most deprived)	1.08 (0.66–1.76) *P* = 0.753
Average public transport travel time to a General Practitioner (GP) surgery in minutes *(categorised into quartiles)*	Q1 (least deprived)	-
	Q2	1.15 (0.71–1.87) *P* = 0.576
	Q3	1.18 (0.73–1.90) *P* = 0.493
	Q4 (most deprived)	1.27 (0.74–2.17) *P* = 0.391
Percentage of people in households that are overcrowded *(categorised into quartiles)*	Q1 (least deprived)	-
	Q2	0.73 (0.44–1.20) *P* = 0.217
	Q3	0.89 (0.55–1.43) *P* = 0.619
	Q4 (most deprived)	1.31 (0.79–2.19) *P* = 0.296

Odds ratios, 95% confidence intervals and *p*-values from univariable, age and sex adjusted, logistic regression analysis for the association between mortality and clinical admission variables, SIMD quintile and 12 selected indicators within the SIMD. For the clinical variables: variables were categorised according to standard reference ranges, with normal values used as the reference. For SIMD quintiles: the least deprived quintile was the reference variable. For the 12 selected SIMD indicators: 1) standardised ratios in the SIMD were transformed into binary variables. Ratios represented observed occurrences divided by the predicted occurrences per datazone, where the reference value was 100, which is the Scotland average for a population with the same age and sex profile. Values above 100 were classed as “ > expected” and values below 100 were “ < expected”. 2) SIMD indicators that were continuous variables (percentages, proportions, or time in minutes) were categorised into quartiles with the least deprived quartile as the reference

**Fig. 3:**
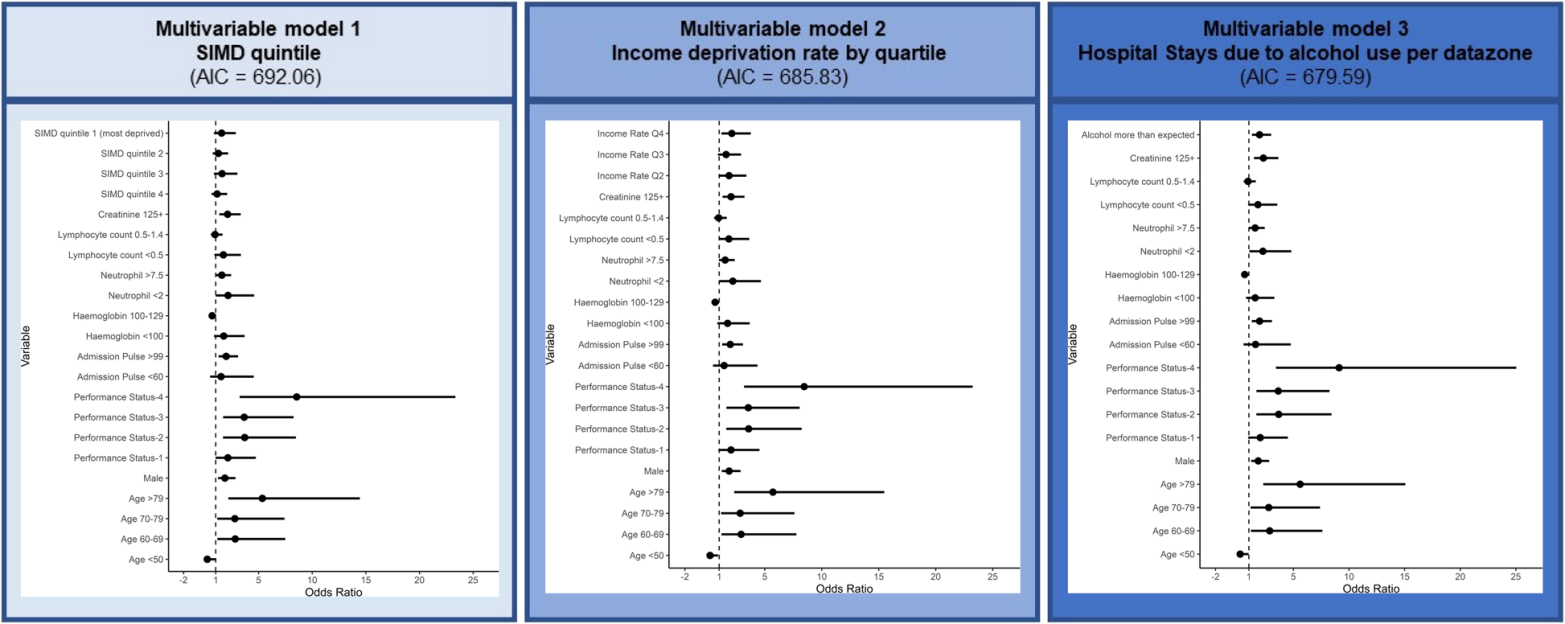
Forest Plots describing multivariable analyses of 3 SIMD indicators associated with increased mortality. Forest plots of odds ratios and 95% confidence intervals from three nested multivariable regression models investigating the association between mortality and: SIMD quintile (model 1); Income deprivation rate by quartile (model 2); Hospital stays due to alcohol use per datazone (model 3). Models were compared for goodness of fit based on Akaike Information Criterion (AIC): SIMD quintile (model 1, AIC: 692.06), income deprivation rate by quartile (model 2, AIC: 685.83), and hospital stays due to alcohol use per datazone (model 3, AIC: 679.59). Each multivariable model also contained: Age (in years), Sex, Performance Status (WHO Standardized Categories), Admission Pulse (beats/minute), Haemoglobin concentration (grams/Litre), Neutrophil count (cells × 10^5^), Lymphocyte count (cells × 10^5^), Creatinine (milligrams/decilitre). These variables were identified at the time of admission as risk variables in a companion paper (*Mutch et al. 2022*) [5]

In our multivariable analysis, we tested associations between mortality and SIMD quintile (Model 1); income deprivation rate per datazone, by quartile (Model 2); and greater than expected hospital stays due to alcohol use per datazone (Model 3) – see Fig. 3.

All three models had an area under the curve of receiver operating characteristic (AUC of ROC) of > 0.8 demonstrating excellent ability to discriminate our primary outcome [18]. Comparison of AIC scores for these nested models demonstrated that Model 2 was the best of the three multivariable models, having the lowest AIC (679.59, vs. Model 3: 685.83, vs. Model 1: 692.06). Detailed results of our multivariable models are available in our supplementary data.

### Comparative distribution of data zones between Lothian and the rest of Scotland

**Fig. 4:**
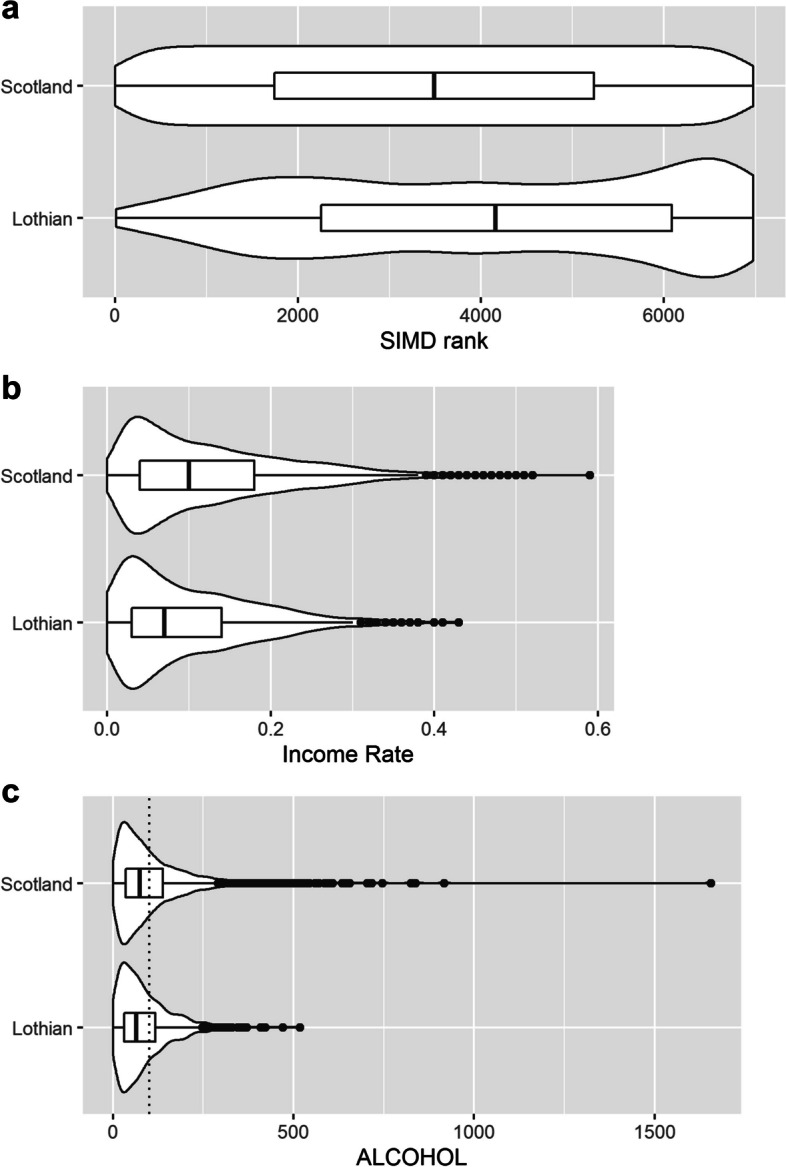
Violin plots. **a** The shape of the distribution of SIMD rank, in Lothian compared to Scotland. SIMD rank of 1 is most deprived and 6976 is least deprived. **b** The shape of the distribution of Income deprivation rate in Lothian compared to Scotland. **c** The shape of the distribution of datazones according to number of hospitalisations related to alcohol; this is a standardised ratio where 100 (dotted line) represents the expected number

We compared the distribution of datazones by SIMD quintile, income deprivation rate, and excess hospital admissions due to alcohol in Lothian and the rest of Scotland (see Fig. 4). SIMD quintile in Lothian demonstrated a bimodal distribution with a greater preponderance of datazones in SIMD quintiles 2 and 5 than the rest of Scotland (see Fig. 4a). We noted a lower median number and smaller distribution range when comparing distribution of datazones by income deprivation rate and greater than expected hospitalisations due to alcohol in the Lothian Region compared to the rest of Scotland (see Fig. 4b and c).

### Sensitivity analysis comparing national and Lothian-specific SIMD quintile distribution

To reflect the known relative affluence of the Lothian region, where up to 50% of residents reside in datazones belonging to SIMD quintiles 1 and 2 (least deprived), we carried out sensitivity analyses using Lothian-specific quintile distributions drawn from publicly available records. We ran Model 1 as described for the SIMD above.

A greater proportion of our patient population was redistributed to more deprived Lothian-specific quintiles, with a greater preponderance of patients in Lothian specific quintiles 1, 2 and 4.

Compared to results obtained using national SIMD quintile distribution, where there were no significant associations with mortality, we found a weak association between mortality and Lothian-specific quintiles 1 and 4 (quintile 1: OR = 2.00 (1.06–3.85) *P* = 0.032; quintile 4: OR = 2.04 (1.06–3.99) *P* = 0.034). The model AIC (Lothian-specific quintile distribution: 689.21) was higher than the model with income deprivation rate per datazone by quartile (Model 2: AIC, 679.59); and greater than expected hospital stays due to alcohol use per datazone (Model 3: AIC 685.83).

## Discussion

In this study, we aimed to establish whether specific indicators of the Scottish Index of Multiple Deprivation (SIMD) were associated with mortality in a prospective cohort of patients admitted to hospital with Covid-19 disease in the Lothian Region between March 1^st^ and June 30^th^, 2020.

Previous studies have demonstrated an increased risk of death in patients living in more deprived communities in multiple countries in the first wave of the Covid-19 pandemic [3, 4, 11–13, 16, 19–21]. We found an increased risk of death among age- and sex-adjusted patients in quintiles 1 and 3 (OR 1.75, CI 0.99–3.08, *P* = 0.053 and OR 2.17, CI 1.22–3.86, *P* = 0.009, respectively), but this association was not upheld in our fully-adjusted multivariable models containing Performance Status and clinical parameters of severity at presentation.

SIMD scores are weighted calculations of each of the seven domains; Income and Employment domain are weighted twice as heavily as Health or Education in final aggregate scores [10]. We therefore selected 12 indicators of deprivation within the SIMD that could plausibly be linked to poorer outcomes in health in our cohort. In our multivariable regression models, patients residing in datazones that were more income deprived and/or reported greater than expected numbers of alcohol-related hospital admissions had a two-fold increased risk of death.

We identified several factors that may explain the divergence in our national SIMD results and contribute to the complexity of defining how deprivation, a multi-faceted entity where environmental, biological, social, economic, and educational factors interact over time, contributes to poorer outcomes in health.

### Overview – deprivation and indicators of multiple deprivation (IMDs)

Deprivation is a well-established risk factor for poorer health outcomes, but its underlying physiological mechanisms remain controversial. Some studies have proposed a biological link whereby increased inflammatory responses triggered by chronic social and environmental stress – more common in deprived communities – accelerate atherosclerosis and progression of dementia [22–24], but few studies have sufficiently long follow-up periods to adequately account for confounders given the multifactorial nature of deprivation [25–28].

Deprivation has also been described as a barrier to accessing healthcare and, in Lothian, this is supported by recent evidence from the Infectious Diseases Outpatient Antibiotic Treatment (OPAT) service that demonstrated that referrals were twice as likely to occur among patients belonging to the least deprived SIMD quintile [29].

Because deprivation is multifactorial, its study relies on amalgamating a range of indicators to develop a detailed picture of residents in a specific location [9, 30, 31]. Indices of multiple deprivation (IMDs) such as the SIMD have gained traction as useful tools for governments to direct funds to specific locations based on the assumption that the spatial characteristics of a geographical locality’s deprivation indicators affects the opportunities for poverty reduction for the entire population [30, 32]. The limitations of this approach are that IMDs fail to capture the key aspects of deprivation affecting any one individual. Experienced general practitioners operating in “Deep End” practices that serve the most deprived communities in Scotland have called for increased devolution of healthcare in at-risk communities as well as heightened awareness of the impact of deprivation on health and health-seeking behaviour to reduce inequities in health [33, 34].

### Study strengths and limitations

We were able to analyse a rich dataset of prospectively recruited individuals benefiting from integration of healthcare data extracted from multiple digital platforms into a centralised database. Our cohort study design enabled us to carry out a detailed analysis of deprivation-related exposures in relation to our outcomes of interest. We believe this is one of the few studies examining the role that specific indicators of deprivation in an IMD may play in contributing to poorer outcomes in patients hospitalised with Covid-19 disease. Whilst we were not able to establish that deprivation by SIMD quintile was a risk factor for poorer outcomes in our cohort, we found that patients who resided in datazones with greater income deprivation and greater-than-expected admissions to hospital due to excess alcohol consumption had a two-fold increased risk of death. This suggests that a more granular analysis of deprivation indicators alongside locally representative deprivation quintile distributions may help to identify individuals or groups at risk of greater mortality in areas where deprivation may be masked by greater overall affluence. This is one of the major strengths of this study.

Our study further highlights the association between income deprivation and increased incidence and higher rates of hospitalization and mortality due to Covid-19, now well-established in both high- and low-income settings, further demonstrating the need for public health interventions to reduce barriers to testing, access to medical services, and mitigation of correlated risk factors for increased mortality such as obesity and co-morbidities [35–38].

Alcohol consumption, has, in contrast, not been found to be significantly associated with poorer outcomes, whether measured in terms of harmful intake in individuals [39] or in spatial analyses of excessive alcohol consumption [40]. In our correlation matrix of our 12 pre-selected SIMD indicators of deprivation, our variable for greater than expected admissions due to alcohol use was strongly associated with comparative illness factor – which measures how many individuals receive contributions for chronic disability – and employment, income, emergency room and drug-related admission rates per datazone. Our findings may reflect the situation in Scotland, where excess hospitalisations and mortality due to harmful alcohol consumption are potentiated by inequality in income, educational attainment, and socio-economic class and may be a useful proxy marker for deprivation not captured elsewhere in the SIMD [41].

Our study has several limitations. First, our study was restricted to hospitalised patients, and we were therefore unable to capture data on community transmission and outcomes in those not admitted to hospital. Another limitation is that a greater proportion of the Lothian region population is both more affluent and less likely to be from a minority ethnic group [42]. In our post-hoc analysis re-running our Model 1 (SIMD quintiles) using Lothian-specific quintile distributions, we found a weak association between mortality and Lothian-specific Quintiles 1 and 4, which in turn mirrored the redistribution of our patient population into locally-representative quintiles. This further highlights the weakness of relying on national SIMD quintile distribution in areas that are less representative of Scotland as a whole. Lastly, other SIMD indicators not selected for logistic regression analysis that our researchers judged less clinically relevant to health outcomes may be strongly influencing SIMD aggregate scores.

Because the Lothian region is comparatively more affluent than other regions of Scotland, it is likely that using postcode-based SIMD as a marker for individual deprivation fails to account for pockets of deprivation in Lothian that are not captured in the traditional quintile distribution of SIMD. The SIMD is an imperfect tool that relies on area-specific characteristics to determine deprivation, and fails to capture non-spatial deprivation factors that contribute to poorer health outcomes among individuals [25, 28]. Further, aggregate scores are weighted according to domain and assign a greater weight to income and employment deprivation than to health. Lastly, SIMD rankings are reviewed based on ten-year census data, which fail to capture between-census demographic change that may influence a specific data-zone’s evolving deprivation ranking, for example, because of gentrification.

Our pilot study highlights interesting findings that shed light on the applicability of SIMD in determining outcomes in patients hospitalised with Covid-19. Our findings may have important policy implications for government responses to targeting public health interventions to address social inequities affecting health outcomes in emerging infectious diseases (EIDs) [43]. We further demonstrate that income deprivation rate and excess hospitalisations due to alcohol use may act as useful proxy indicators to identify areas of Scotland where these social inequities are not adequately captured by aggregate SIMD ranking. We plan to apply our model to a nationwide dataset to determine whether these SIMD indicators may be applicable at a national level and in the context of future responses to EIDs.

## Conclusions

We present findings of a prospective cohort study of patients hospitalised with Covid-19 in the Lothian region recruited consecutively during the first wave of the Covid-19 pandemic. We performed unadjusted and age- and sex-adjusted univariable analysis and compared three multivariable models investigating the impact of aggregate and specific indicators of deprivation on mortality. We found that locally representative SIMD quintile distribution and, within specific indicators of deprivation, datazones that were more income deprived and those with greater than expected number of hospitalisations due to alcohol use were associated with an increased risk of death. In contrast to other studies, greater deprivation as measured by national SIMD quintile distribution was not associated with mortality in our cohort. We propose that our findings are divergent due to the demographic characteristics of the Lothian population, which is generally more affluent and ethnically homogenous than the wider Scottish population and where up to 50% of deprived individuals live in non-deprived datazones [32]. Lastly, we suggest that further research could investigate how individual indicators of deprivation may help target future government response to EIDs and identify population subgroups at risk of poorer health outcomes not captured by SIMD quintile.

### Supplementary Information

**Additional file 1.** Multivariable logistic regression analysis between mortality and three SIMD indicators carried forward from univariable analysis.

## Data Availability

The data that support the findings of this study are available from DataLoch (Edinburgh, United Kingdom) but restrictions apply to the availability of these data, which were used under license for the current study, and so are not publicly available. Data are however available from the authors upon reasonable request and with permission of DataLoch.
