# Supplementary material for: Scottish Index of Multiple Deprivation (SIMD) indicators as predictors of mortality among patients hospitalised with COVID-19 disease in the Lothian Region, Scotland during the first wave: a cohort study
Source: Int J Equity Health. 2023 Oct 5;22:205. doi: 10.1186/s12939-023-02017-y (PMC10552319; doi:10.1186/s12939-023-02017-y)
Supplement: Supplementary file 1 — Additional file 1. Multivariable logistic regression analysis between mortality and three SIMD indicators carried forward from univariable analysis. [file 12939_2023_2017_MOESM1_ESM.docx]

# Supplementary Material

**Supplementary Material – Multivariable logistic regression analysis between mortality and three SIMD indicators carried forward from univariable analysis.**

| **Variables**  ***(units)*** | **Variable ranges** | **Multivariable Model 1**  **SIMD quintile**  **(Odds Ratios, 95% CI)** | **Multivariable Model 2**  **Income Deprivation Rate, by quartile**  **(Odds Ratios, 95% CI)** | **Multivariable Model 3**  **Hospital stays due to alcohol use per datazone**  **(Odds Ratios, 95% CI)** |
| --- | --- | --- | --- | --- |
| **Age brackets**  *(in years)* | 50-59 | 1 | 1 | 1 |
|  | <50 | 0.21 (0.03-0.94) *P=*0.064 | 0.20 (0.03-0.91) *P=*0.058 | 0.22 (0.03-0.98) *P=*0.070 |
|  | 60-69 | 2.82 (1.15-7.48) *P=*0.028 | 2.93 (1.20-7.77) *P=*0.023 | 2.87 (1.18-7.61) *P=*0.025 |
|  | 70-79 | 2.78 (1.15-7.41) *P=*0.030 | 2.85 (1.17-7.61) *P=*0.027 | 2.79 (1.15-7.40) *P=*0.029 |
|  | >79 | 5.35 (2.18-14.44) *P*<0.001 | 5.72 (2.33-15.49) *P*<0.001 | 5.61 (2.30-15.07) *P*<0.001 |
| **Sex** | Female | 1 | 1 | 1 |
|  | Male | 1.85 (1.22-2.84) *P=*0.004 | 1.87 (1.23-2.88) *P=*0.004 | 1.83 (1.20-2.82) *P=*0.005 |
| **Performance Status**  *(WHO Standardized Categories)* | 0 | 1 | 1 | 1 |
|  | 1 | 2.13 (0.99-4.73) *P=*0.058 | 2.04 (0.94-4.5) *P=*0.073 | 2.02 (0.94-4.50) *P=*0.077 |
|  | 2 | 3.70 (1.68-8.47) *P=*0.001 | 3.59 (1.62-8.24) *P=*0.002 | 3.68 (1.67-8.43) *P=*0.002 |
|  | 3 | 3.65 (1.69-8.25) *P=*0.001 | 3.56 (1.64-8.06) *P=*0.002 | 3.64 (1.68-8.25) *P=*0.001 |
|  | 4 | 8.55 (3.24-23.34) *P*<0.001 | 8.46 (3.18-23.24) *P*<0.001 | 9.11 (3.43-25.04) *P*<0.001 |
| **Heart rate on admission**  *(beats per minute)* | 60-99 | 1 | 1 | 1 |
|  | <60 | 1.49 (0.48-4.54) *P=*0.480 | 1.44 (0.46-4.36) *P=*0.521 | 1.60 (0.52-4.77) *P=*0.400 |
|  | >99 | 1.98 (1.27-3.08) *P=*0.003 | 1.97 (1.27-3.09) *P=*0.003 | 1.96 (1.26-3.07) *P=*0.003 |
| **Haemoglobin concentration**  *(grams/Litre)* | >129 | 1 | 1 | 1 |
|  | <100 | 1.74 (0.84-3.69) *P=*0.140 | 1.74 (0.83-3.68) *P=*0.141 | 1.56 (0.75-3.30) *P=*0.234 |
|  | 100-129 | 0.66 (0.42-1.03) *P=*0.072 | 0.65 (0.41-1.00) *P=*0.054 | 0.62 (0.40-0.97) *P=*0.040 |
| **Neutrophil count**  *(cells x 10^5^)* | 2-7.5 | 1 | 1 | 1 |
|  | <2 | 2.15 (1.00-4.58) *P=*0.049 | 2.20 (1.02-4.66) *P=*0.041 | 2.26 (1.05-4.82) *P=*0.035 |
|  | >7.5 | 1.57 (1.01-2.44) *P=*0.045 | 1.52 (0.98-2.37) *P=*0.062 | 1.56 (1.00-2.42) *P=*0.048 |
| **Lymphocyte count**  *(cells x 10^5^)* | >1.4 | 1 | 1 | 1 |
|  | <0.5 | 1.71 (0.88-3.35) *P=*0.114 | 1.85 (0.95-3.64) *P=*0.070 | 1.81 (0.94-3.55) *P=*0.080 |
|  | 0.5-1.4 | 0.94 (0.55-1.63) *P=*0.816 | 0.95 (0.55-1.66) *P=*0.864 | 0.92 (0.53-1.61) *P=*0.767 |
| **Creatinine**  *(milligrams/decilitre)* | <125 | 1 | 1 | 1 |
|  | 125+ | 2.11 (1.34-3.32) *P=*0.001 | 2.04 (1.30-3.22) *P=*0.002 | 2.30 (1.46-3.65) *P*<0.001 |
| **SIMD quintile** | 5 (least deprived) | 1 (ref) |  |  |
|  | 4 | 1.13 (0.61-2.07) *P=*0.692 |  |  |
|  | 3 | 1.59 (0.84-3.02) *P=*0.155 |  |  |
|  | 2 | 1.25 (0.72-2.16) *P=*0.425 |  |  |
|  | 1 (most deprived) | 1.55 (0.84-2.87) *P=*0.162 |  |  |
| **Income Deprivation Rate**  *(categorised by quartile)* | Q1 (least deprived) |  | 1 (ref) |  |
|  | Q2 |  | 1.87 (1.04-3.39) *P=*0.038 |  |
|  | Q3 |  | 1.61 (0.90-2.92) *P=*0.114 |  |
|  | Q4 (most deprived) |  | 2.11 (1.20-3.77) *P=*0.011 |  |
| **Hospital stays due to alcohol use per datazone**  *(Standardised Ratio*)* | < expected |  |  | 1 (ref) |
|  | > expected |  |  | 1.96 (1.28-3.00) *P=*0.002 |

**Legend:** Odds ratios, 95% confidence intervals, and p-values from three nested multivariable models: SIMD quintile (**Model 1**, AIC: 692.06), income deprivation rate (**Model 2**, AIC: 685.83), and hospital admissions due to alcohol use (**Model 3**, AIC: 679.59). Standardised ratios in the SIMD were transformed into binary variables. Ratios represented observed occurrences divided by the predicted occurrences per datazone, where the reference value was 100, which is the Scotland average for a population with the same age and sex profile. SIMD indicators that were continuous variables (percentages, proportions, or time in minutes) were categorised into quartiles with the least deprived quartile as the reference. Each multivariable model also had age, sex, Performance Status and clinical parameters at the time of admission as variables.
